# Protective Role of Thiamine Pyrophosphate Against Erlotinib-Induced Oxidative and Inflammatory Damage in Rat Optic Nerve

**DOI:** 10.3390/biomedicines13112614

**Published:** 2025-10-25

**Authors:** Ezgi Karatas, Bulent Yavuzer, Ozlem Demir, Esra Tuba Sezgin, Engin Hendem, Emine Cinici, Taha Abdulkadir Coban, Halis Suleyman

**Affiliations:** 1 Department of Ophthalmology, Faculty of Medicine, Agrı Ibrahim Cecen University, Agrı 04100, Turkey; ezkaratas@agri.edu.tr; 2 Department of Pharmacology, Faculty of Medicine, Erzincan Binali Yıldırım University, Erzincan 24100, Turkey; bulent.yavuzer@erzincan.edu.tr; 3 Department of Histology and Embryology, Faculty of Medicine, Erzincan Binali Yıldırım University, Erzincan 24100, Turkey; ozlem.abuc@erzincan.edu.tr; 4 Anesthesia Program, Vocational School of Health Services, Erzincan Binali Yıldırım University, Erzincan 24036, Turkey; esra.demir@erzincan.edu.tr; 5 Department of Medical Oncology, Mengucek Gazi Education and Research Hospital, Erzincan Binali Yıldırım University, Erzincan 24100, Turkey; enginhende61@gmail.com; 6 Department of Ophthalmology, Faculty of Medicine, Ataturk University, Erzurum 25240, Turkey; emine.cinici@atauni.edu.tr; 7 Department of Medical Biochemistry, Faculty of Medicine, Erzincan Binali Yıldırım University, Erzincan 24100, Turkey; acoban@erzincan.edu.tr

**Keywords:** erlotinib, inflammation, neuroprotection, optic nerve, oxidative stress, protein kinase inhibitors, rats, reactive oxygen species, receptor, epidermal growth factor, thiamine pyrophosphate

## Abstract

**Background:** Epidermal growth factor receptor tyrosine kinase inhibitors (EGFR-TKIs) such as erlotinib are widely used in non-small-cell lung cancer treatment, and accumulating evidence indicates they can markedly increase ocular toxicity. Nonetheless, whether erlotinib causes optic nerve injury has not been investigated before and remains a subject worth investigating. This study aimed to examine the impact of erlotinib on oxidative stress, inflammation, and histopathological changes in rat optic nerve tissue and evaluate the potential neuroprotective role of thiamine pyrophosphate (TPP). **Methods:** Twenty-four male Wistar rats were randomly assigned to four groups: healthy control, TPP alone, erlotinib alone, and erlotinib + TPP. Erlotinib (10 mg/kg, orally, on alternate days) and TPP (20 mg/kg, intraperitoneally, daily) were administered for two consecutive weeks. Optic nerve samples were analyzed for malondialdehyde (MDA), total glutathione (tGSH), superoxide dismutase (SOD), catalase (CAT), interleukin-1β (IL-1β), and tumor necrosis factor-α (TNF-α), followed by histopathological examination. **Results:** Erlotinib treatment significantly increased MDA, IL-1β, and TNF-α levels while reducing tGSH, SOD, and CAT activity, demonstrating oxidative stress and an inflammatory response. Co-administration of TPP ameliorated these changes by lowering reactive oxygen species, restoring antioxidant capacity, and attenuating inflammation. Histopathological alterations, including astrocyte degeneration, edema, and vascular congestion, were evident after erlotinib exposure but were significantly alleviated when TPP was administered concurrently. **Conclusions:** Erlotinib induces oxidative and inflammatory optic nerve injury, while TPP co-treatment offers significant neuroprotection. These findings support TPP as a potential adjunct to reduce EGFR-TKI-related ocular toxicity and highlight importance of redox modulation in limiting treatment-associated side effects.

## 1. Introduction

Erlotinib is an orally administered reversible tyrosine kinase inhibitor (TKI) that targets the epidermal growth factor receptor (EGFR) [1]. EGFR is a receptor that plays a pivotal role in maintaining the structural integrity and functional capacity of epithelial tissues [2]. EGFR-TKIs are recommended as the standard first-line therapy for non-small-cell lung cancer (NSCLC) [3,4]. TKIs have become a turning point in targeted therapy strategies due to their low incidence of side effects. Four generations of TKIs have been developed to overcome resistance caused by EGFR mutations [5]. Erlotinib is a first-generation TKI group that competes with adenosine-5′-triphosphate (ATP) to bind to the intracellular tyrosine kinase domain of the receptor [6,7,8]. EGFR activation contributes to wound healing and preservation of the epidermal barrier, whereas its suppression may reduce epidermal thickness. This results in numerous side effects associated with EGFR-TKI therapy [9]. Skin dryness, erythema, pruritus, and paronychia are among the adverse effects of this treatment [10,11]. Ocular adverse effects associated with erlotinib may vary from mild dry eye to severe disorders, including corneal perforation [12,13]. EGFR plays a critical role in corneal regeneration, in maintaining the proliferative capacity of epithelial cells, and in regulating hair follicle differentiation. Consequently, EGFR inhibition may result in dry eye, keratitis, conjunctivitis, blepharitis, episcleritis, ectropion, entropion, and eyelash trichomegaly [14,15]. Case reports have also identified uveitis as a potential ocular toxicity associated with erlotinib [16,17,18]. A case of bilateral retinochoroiditis resulting from toxoplasmosis following erlotinib therapy has been reported in the literature [19]. Certain side effects of erlotinib are associated with elevated levels of reactive oxygen species (ROS) [20,21,22,23]. Several retinal disorders that may occur during erlotinib therapy, such as glaucoma and age-related macular degeneration, are directly associated with elevated levels of ROS. Increased ROS production is known to trigger processes including inflammation, retinal vascular endothelial dysfunction, and neuronal degeneration [24].

Thiamine pyrophosphate (TPP) represents the biologically active coenzyme form of thiamine (vitamin B1). Thiamine is prevalent in the liver, semolina, yeast, meat, and whole grain products [25]. Thiamine pyrophosphokinase converts thiamine into its active form, TPP, within the body [26]. TPP serves as a cofactor for pyruvate dehydrogenase, which catalyzes the conversion of pyruvate to acetyl coenzyme A under aerobic conditions [27]. In TPP deficiency, pyruvate is converted to lactate, potentially leading to lactic acidosis due to lactic acid accumulation [28].

It has been emphasized in previous studies that thiamine deficiency is strongly linked to oxidative stress, which in turn contributes critically to the onset of neurodegenerative changes [29,30]. Chauhan et al. demonstrated that thiamine deficiency disrupts energy metabolism, leading to increased lipid peroxidation (LPO) and a marked reduction in the levels of enzymes involved in the antioxidant defense system. Reports indicate that this situation is linked to acute oxidative stress, impairment of mitochondrial function, and neuronal injury [31]. The literature indicates that TPP protects the optic nerve from oxidative and inflammatory injury by preventing ethanol-induced increases in malondialdehyde (MDA), interleukin-1 beta (IL-1β), and tumor necrosis factor-alpha (TNF-α) levels, as well as decreases in glutathione (GSH), superoxide dismutase (SOD), and catalase (CAT) [32]. Previous studies have reported that thiamine’s active metabolite, TPP, plays a fundamental role in cellular energy metabolism [33]. These findings suggest that TPP may be effective in counteracting potential optic nerve damage induced by erlotinib. To date, the literature contains no experimental investigations into the effects of erlotinib on optic nerve tissue. Furthermore, the literature provides no evidence regarding the possible role of TPP in protecting against optic nerve damage associated with erlotinib. Therefore, the aim of our study is to investigate the optic nerve tissues of rats treated with erlotinib through biochemical and histopathological analyses, and to evaluate the potential protective effect of TPP against erlotinib-induced optic nerve damage.

## 2. Materials and Methods

### 2.1. Animals

In this experimental study, 24 male albino Wistar-type rats, aged 9–10 weeks with body weights ranging from 280 to 287 g, were employed. All subjects were obtained from Erzincan Binali Yıldırım University’s Experimental Animals Application and Research Center (Erzincan, Turkey). Rats were randomly assigned into four groups of six animals each, ensuring comparable mean body weights across groups. Before the experiment, during the acclimatization period, the rats were kept in four standard wire laboratory cages (20 cm × 35 cm × 55 cm; floor area: 1925 cm^2^), with six animals housed per cage. All subjects were maintained under regulated conditions, including a 12 h/12 h light-dark photoperiod, stable ambient temperature (22 ± 2 °C), and 30–70% relative humidity. During the course of the study, rats had unrestricted access to tap water and a standard laboratory chow (Bayramoglu Stock Company, Erzurum, Turkey). All procedures involving animals were conducted in the laboratories of the Experimental Animal Application and Research Center at Erzincan Binali Yıldırım University.

This study was conducted in compliance with the European Directive 2010/63/EU for the protection of animals used in scientific research (Approval ID: 2016-24-199) and adhered to the ARRIVE (Animal Research: Reporting of In Vivo Experiments) guidelines [34]. 

### 2.2. Chemicals

In this study, thiopental sodium (Pental Sodyum^®^, 0.5 g vial) was purchased from Menarini Health and Pharmaceuticals Industry Trade Inc. (Istanbul, Turkey), erlotinib (Tarceva^®^, 100 mg tablet) was obtained from Roche Pharmaceuticals Industry Inc. (Istanbul, Turkey) and thiamine pyrophosphate (Cocarboxylase hydrochloride^®^, 50 mg/2 mL injectable solution) was supplied by BioPharma (Kiev, Ukraine).

### 2.3. Experimental Design and Grouping

#### 2.3.1. Experimental Design

In accordance with the 4R principles (Reduction, Refinement, Replacement, and Responsibility) [35], the sample size was determined to ensure the minimum necessary number of animals. Animals showing signs such as hunched posture, decreased mobility, or injuries from cage mates were considered for exclusion from the study and analysis, though none were excluded. Randomization was carried out using a random number table, and numerical labeling was applied to both cages and animals to reduce potential confounding factors.

#### 2.3.2. Experimental Groups

Animals were randomly allocated into four experimental groups: healthy control group (HG), TPP alone group (TPPG), erlotinib alone group (ERTG), and erlotinib combined with TPP group (ERTPG).

### 2.4. Experiment Procedure

TPP was administered intraperitoneally at 20 mg/kg to the TPPG (*n* = 6) and ERTPG (*n* = 6) groups, while the ERTG (*n* = 6) and HG (*n* = 6) groups received saline (0.9% NaCl) as vehicle by the same route. Following a one-hour interval, erlotinib (10 mg/kg) was administered orally by gavage to rats in the ERTG and ERTPG. TPP was applied daily, while erlotinib was administered on alternate days over a two-week period. At the end of the experimental protocol, animals were euthanized under deep anesthesia induced with thiopental sodium (50 mg/kg), and optic nerve tissues were harvested. Biochemical assays were performed to determine MDA, GSH, SOD, CAT, IL-1β, and TNF-α levels, and histopathological analyses of optic nerve tissue were also carried out. The experimental outcomes from all groups were then comparatively assessed.

### 2.5. Biochemical Analysis

#### 2.5.1. Preparation of Samples

Approximately 10 mg of optic nerve tissue was dissected from each rat and rinsed with 0.9% sodium chloride solution to eliminate blood and debris. Following homogenization under ice-cold conditions with a high-speed tissue homogenizer, 2 mL of 1.15% potassium chloride (KCl) buffer (pH 7.4) was added to each sample. The homogenates were centrifuged at 10,000× *g* for 15 min at 4 °C, and the resulting supernatants were collected for biochemical analysis. From these fractions, MDA and tGSH levels, as well as the enzymatic activities of SOD and CAT, were quantified. To ensure comparability between groups, all biochemical measurements were normalized to total protein content and expressed as milligrams per gram of protein (mg/g protein).

#### 2.5.2. Determination of MDA, tGSH, SOD, CAT, and Protein in Optic Nerve Tissue

Levels of MDA, tGSH, and SOD were quantified using rat-specific ELISA kits (MDA: #10009055; tGSH: #703002; SOD: #706002; Cayman Chemical, Ann Arbor, MI, USA) in accordance with the protocols provided by the manufacturer. The CAT determination was conducted following the methodology suggested by Goth [36]. Protein concentrations were evaluated using the Bradford assay, which relies on the binding of Coomassie Brilliant Blue G-250 dye to proteins, with absorbance measured spectrophotometrically at 595 nm [37].

#### 2.5.3. Determination of IL-1β and TNF-α Levels in Optic Nerve Tissue

The tissue samples were first weighed, then cut into small pieces, rapidly frozen in liquid nitrogen, and homogenized using a pestle and mortar. After thawing, the homogenates were maintained at 2–8 °C. Phosphate-buffered saline (PBS, pH 7.4) was added at a ratio of 1:10 (*w*/*v*), followed by vortexing for 10 s. The mixtures were centrifuged at 10,000× *g* for 20 min, and the supernatants were carefully collected. Levels of TNF-α (ng/L) and IL-1β (pg/L) were quantified using commercial ELISA kits (Hangzhou Eastbiopharm Co., Ltd., Hangzhou, China) according to the manufacturer’s instructions.

### 2.6. Histopathological Analysis

All optic nerve tissue samples were initially fixed in a 10% formaldehyde solution for light microscopic evaluation. Following fixation, the tissue samples were rinsed in tap water in cassettes for 24 h. Dehydration was then carried out using conventional grades of alcohol (70%, 80%, 90%, and 100%), after which tissues were cleared in xylol and embedded in paraffin. Sections measuring four–five microns were excised from paraffin blocks and subjected to hematoxylin–eosin staining. Serial sections were prepared from optic nerve tissues of six animals per group (*n* = 6). For each section, one central area and five peripheral fields were examined under 400× magnification, yielding 36 images evaluated for each group. Photomicrographs were captured using the Olympus DP2-SAL firmware program (Olympus Inc.^®^, Tokyo, Japan). Histopathological changes in optic nerve tissue were characterized by increase in astrocyte cell population, edema, and vascular dilatation or congestion. Tissue injury was scored on a four-point scale: 0 = no damage, 1 = mild, 2 = moderate, and 3 = severe [38,39,40]. All histopathological evaluations were conducted by a pathologist blinded to group assignments.

### 2.7. Statistical Analysis

Statistical analyses of biochemical and histopathological parameters were performed using IBM SPSS Statistics for Windows (Version 27.0; IBM Corp., Armonk, NY, USA, 2020). Graphical illustrations were generated with GraphPad Prism (Version 8.0.1; GraphPad Software, San Diego, CA, USA, 2018). Biochemical results are presented as mean ± standard error of the mean (SEM). The distribution of the data was examined with the Shapiro–Wilk test, and variance homogeneity was evaluated using Levene’s test (Supplementary Tables S1 and S2). Intergroup comparisons were conducted using one-way ANOVA, followed by Tukey’s honestly significant difference (HSD) test for post hoc pairwise analyses. Histopathological findings are expressed as median values with corresponding minimum and maximum ranges. Differences in histopathological scores between groups were assessed using the nonparametric Kruskal–Wallis test. For post hoc multiple comparisons, Dunn’s test with Bonferroni correction was applied, and adjusted *p*-values are reported. A threshold of *p* < 0.05 was considered statistically significant.

## 3. Results

### 3.1. Biochemical Findings

#### 3.1.1. The Outcomes of the MDA and tGSH Assays in Optic Nerve Tissue

**Figure 1:**
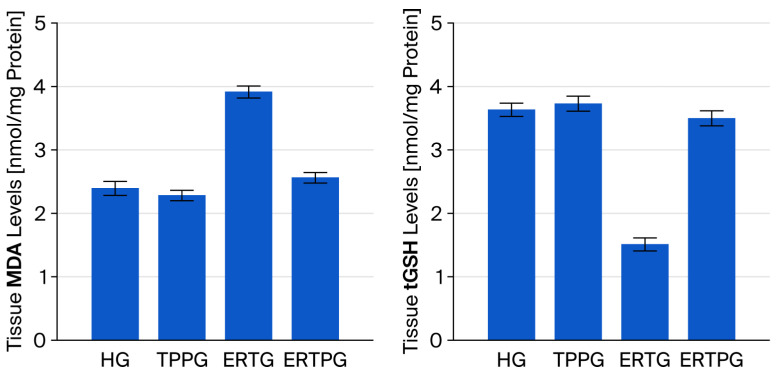
Impact of thiamine pyrophosphate and erlotinib administration on MDA and tGSH levels in rat optic nerve tissue. **Footnotes:** Values are expressed as mean ± SEM (standard error of the mean). **Abbreviations:** HG: healthy group; TPPG: TPP alone group; ERTG: erlotinib alone group; ERTPG: erlotinib + TPP group; TPP: thiamine pyrophosphate; MDA: malondialdehyde; tGSH: total glutathione.

As illustrated in Figure 1 and Table 1, the mean MDA level in the optic nerve tissue of the animals treated with TPP alone (TPPG, 2.29 ± 0.08) was comparable to that of the healthy control group (HG, 2.40 ± 0.11), and the difference did not reach statistical significance (*p* = 0.847). In contrast, the group administered erlotinib alone (ERTG, 3.92 ± 0.10) exhibited markedly elevated MDA levels relative to both the healthy controls and the TPP-only group (both *p* < 0.001). Notably, co-treatment with TPP (ERTPG, 2.57 ± 0.09) significantly prevented the erlotinib-induced increase in MDA levels (*p* < 0.001), with values remaining close to those observed in the healthy controls (*p* = 0.607).

In the TPP-treated group (TPPG, 3.72 ± 0.12), the optic nerve tissue tGSH level was nearly identical to that of the healthy controls (HG, 3.62 ± 0.11), and the difference was not statistically significant (*p* = 0.933). By contrast, animals exposed to erlotinib alone (ERTG, 1.51 ± 0.10) exhibited a profound reduction in tGSH levels when compared with both the healthy and TPP-only groups (*p* < 0.001 for each comparison). Administration of TPP together with erlotinib (ERTPG, 3.49 ± 0.12) effectively counteracted this depletion, yielding values significantly higher than those of the ERTG (*p* < 0.001) and closely approximating those of the healthy controls (*p* = 0.834) (Figure 1 and Table 1).

#### 3.1.2. The Outcomes of the SOD and CAT Assays in Optic Nerve Tissue

**Figure 2:**
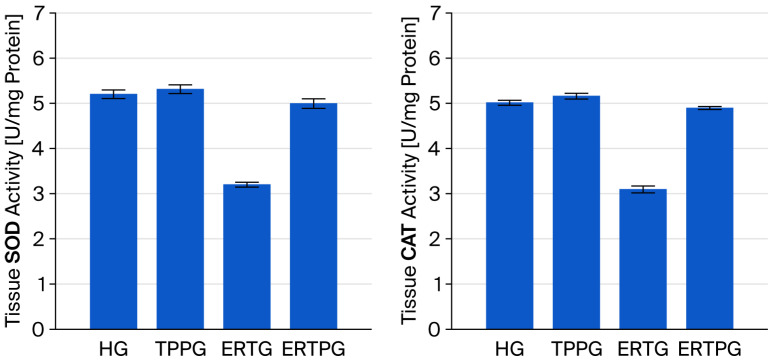
Effects of thiamine pyrophosphate and erlotinib on SOD and CAT activity in rat optic nerve tissue. **Footnotes:** Values are expressed as mean ± SEM (standard error of the mean). **Abbreviations:** HG: healthy group; TPPG: TPP alone group; TPP: thiamine pyrophosphate; ERTG: erlotinib alone group; ERTPG: erlotinib + TPP group; SOD: superoxide dismutase; CAT: catalase.

As presented in Figure 2 and Table 1, SOD activity in the optic nerve tissue of the TPP-only group (TPPG, 5.32 ± 0.10) was comparable to that observed in the healthy controls (HG, 5.21 ± 0.10), with no significant difference between the groups (*p* = 0.824). In contrast, animals administered erlotinib alone (ERTG, 3.20 ± 0.05) displayed markedly reduced SOD activity compared with both the healthy and TPP groups (*p* < 0.001 for each). Importantly, co-administration of TPP (ERTPG, 5.00 ± 0.11) significantly prevented the erlotinib-related decline in SOD activity (*p* < 0.001), and the values in this group were not statistically different from those of the healthy controls (*p* = 0.415).

The CAT activity measured in the optic nerve tissue of the TPP-only animals (TPPG, 5.17 ± 0.07) was essentially equivalent to that of the healthy controls (HG, 5.02 ± 0.06), with no statistically significant variation (*p* = 0.367). Conversely, erlotinib treatment alone (ERTG, 3.10 ± 0.08) resulted in a pronounced reduction in CAT activity relative to both the healthy and TPP groups (*p* < 0.001 for each comparison). Co-treatment with TPP (ERTPG, 4.90 ± 0.04) markedly attenuated this reduction (*p* < 0.001), and the levels recorded in this group remained statistically indistinguishable from those of the healthy controls (*p* = 0.579) (Figure 2 and Table 1).

#### 3.1.3. The Outcomes of the IL-1β and TNF-α Assays in Optic Nerve Tissue

**Figure 3:**
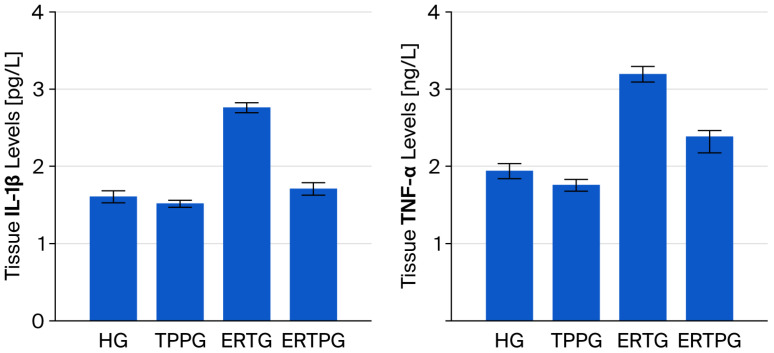
Impact of thiamine pyrophosphate and erlotinib administration on IL-1β and TNF-α levels in rat optic nerve tissue. **Footnotes:** Values are expressed as mean ± SEM (standard error of the mean). **Abbreviations:** HG: healthy group; TPPG: TPP alone group; TPP: thiamine pyrophosphate; ERTG: erlotinib alone group; ERTPG: erlotinib + TPP group; IL-1β: interleukin-1 beta; TNF-α: tumor necrosis factor-alpha.

According to the data presented in Figure 3 and Table 1, IL-1β levels in the optic nerve tissue of the TPP-only group (TPPG, 1.52 ± 0.05) were nearly identical to those in the healthy controls (HG, 1.61 ± 0.08), with no significant difference detected (*p* = 0.806). By contrast, animals that received erlotinib alone (ERTG, 2.76 ± 0.07) showed a substantial rise in IL-1β levels when compared with both the healthy and TPP-treated groups (*p* < 0.001 for both). Administration of TPP together with erlotinib (ERTPG, 1.71 ± 0.08) significantly blunted this increase (*p* < 0.001), and the values in this group remained statistically indistinguishable from those of the healthy controls (*p* = 0.743).

TNF-α levels in the optic nerve tissue of animals treated solely with TPP (TPPG, 1.76 ± 0.08) were closely aligned with those of the healthy controls (HG, 1.94 ± 0.10), and the difference was not statistically significant (*p* = 0.764). In contrast, erlotinib administration alone (ERTG, 3.20 ± 0.10) produced a pronounced elevation in TNF-α levelscompared with both the healthy and TPP-only groups (*p* < 0.001 for each). When TPP was co-administered with erlotinib (ERTPG, 2.39 ± 0.21), this rise was significantly mitigated (*p* = 0.002), yielding values that did not differ significantly from those of the healthy controls (*p* = 0.117) (Figure 3 and Table 1).

### 3.2. Histopathological Findings

**Figure 4:**
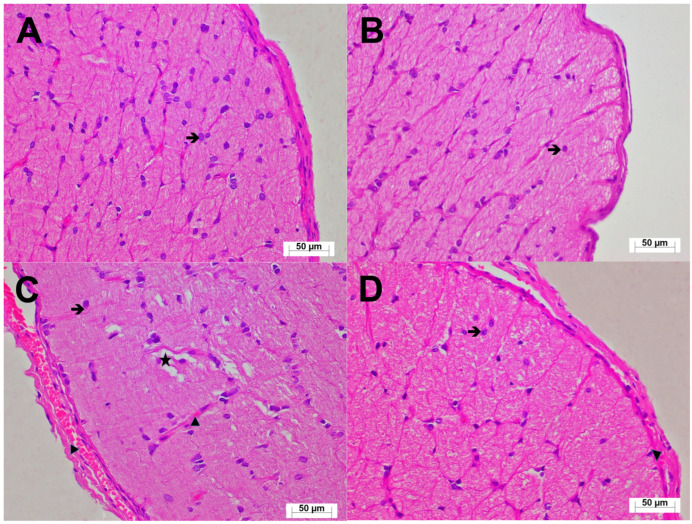
(**A**) Optic nerve tissue from the HG showing normal histological architecture (H&E, ×400; 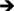: astrocyte). (**B**) Optic nerve tissue from the TPPG with preserved histological appearance (H&E, ×400; 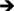: astrocyte). (**C**) Optic nerve tissue from the ERTG demonstrating increased hypertrophic/degenerative astrocytes (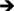), marked edema (★), pronounced vascular congestion (▶: blood capillary) (H&E, ×400). (**D**) Optic nerve tissue from the ERTPG showing nearly normal histological appearance with astrocytes of normal morphology (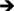) and occasional vascular congestion (▶: blood capillary) (H&E, ×400). **Abbreviations:** HG: healthy group; TPPG: TPP alone group; TPP: thiamine pyrophosphate; ERTG: erlotinib alone group; ERTPG: erlotinib + TPP group.

Histological evaluation of the optic nerve tissue under light microscopy from the healthy controls (HG; Figure 4A) and the animals treated with TPP alone (TPPG; Figure 4B) showed preserved microscopic architecture of the connective tissue and trabeculae surrounding the optic nerve. No pathological changes were observed in the blood vessels or astrocytes. In contrast, optic nerve sections from the erlotinib-treated group (ERTG) displayed areas of edema, separations in the surrounding connective tissue, and congestion within dilated blood vessels. An increased number of hypertrophic and degenerative astrocytes was clearly observed (Figure 4C). In the group receiving erlotinib in combination with TPP (ERTPG), the histological appearance of the optic nerve closely resembled that of the healthy controls. The surrounding connective tissue and astrocytes appeared normal, with only rare vascular congestion noted in the connective tissue adjacent to the optic nerve (Figure 4D). The semi-quantitative histopathological findings and the results of multiple comparisons are presented in Table 2.

## 4. Discussion

In this study, the potential protective role of thiamine pyrophosphate (TPP) against erlotinib-induced optic nerve injury was investigated through both biochemical and histopathological methods. Biochemical analyses indicated that erlotinib treatment resulted in elevated levels of oxidants (MDA) and inflammatory markers (IL-1β and TNF-α), alongside a reduction in antioxidants (tGSH, SOD, and CAT). Our findings demonstrate that TPP attenuates erlotinib-associated oxidative and inflammatory responses while concurrently enhancing the antioxidant defense system. It is well recognized that cells possess a variety of antioxidant defense systems to regulate and counterbalance the production of ROS [41]. An imbalance between ROS production and cellular antioxidant defense mechanisms can lead to oxidative damage and inflammation [42,43,44]. The eye is an organ that is highly sensitive to oxidative stress due to its elevated metabolic activity [45]. It has been reported that damage mediated by ROS is closely associated with the pathogenesis of various ocular diseases [46]. Elevated ROS production induces various processes, including inflammation, dysfunction of retinal vascular endothelial cells, and neuronal degeneration [24]. The measurement of MDA levels serves as a crucial biomarker for assessing oxidative stress and detecting ROS-induced damage in both in vivo and in vitro contexts [47,48,49]. In a study involving TKIs, including erlotinib, it was emphasized that elevated ROS levels damage DNA, proteins, and organelles, thereby contributing to cancer cell death [50]. Our study showed that MDA levels in the optic nerve tissues of animals treated solely with erlotinib were significantly elevated compared to those in the healthy control group, TPPG, and ERTPG. The present data confirm the oxidative stress-inducing effect of erlotinib, aligning with prior evidence from the literature [21]. Our results, in agreement with earlier studies, demonstrated that erlotinib administration alone significantly decreased tGSH, SOD, and CAT levels, all of which are essential elements of the antioxidant defense system [51]. Ozawa et al. reported that antioxidant agents exhibit tissue protective effects against retinal oxidative damage [52].

It is well established that oxidative stress plays a pivotal role in the pathogenesis of degenerative, genetic, age-associated, and inflammatory eye diseases [53]. TNF-α, a proinflammatory cytokine, has been implicated in systemic inflammation [54,55]. Tezel et al. pretreated optic nerve head glial cells with ROS and observed a release of TNF-α that was approximately six times greater than that of normal glia [56]. IL-1β is recognized for its role in regulating the expression of multiple inflammatory molecules [57]. Tamer et al. indicated that proinflammatory cytokines, including TNF-α and IL-1β, are responsible for inducing cell death and chronic inflammation [58]. Our study revealed elevated proinflammatory cytokine levels in the optic nerve tissues of erlotinib-treated rats compared to those in the healthy control group, TPPG, and ERTPG.

The antioxidant TPP, which we examined for its effect on erlotinib-induced optic nerve damage, is important for sustaining normal levels of cell metabolism. Consequently, deficiency leads to disrupted oxidative metabolism, resulting in the onset of neurodegenerative diseases [31]. The elevation of MDA, IL-1β, and TNF-α levels, along with the reduction in tGSH, SOD, and CAT levels resulting from erlotinib administration, was suppressed in the TPP group. Our findings are consistent with those of Ucak et al. and support a protective effect of TPP against oxidative and inflammatory injury to the optic nerve [32]. The research conducted by Turan et al. examined the effect of TPP on cisplatin-induced liver injury and demonstrated that TPP protected against oxidative stress [59]. The findings of a study by Yeter et al. which examined the protective role of TPP in drug-induced liver injury, suggest that TPP mitigates oxidative damage [60].

Histopathological findings were concordant with the biochemical data. The group receiving erlotinib alone exhibited connective tissue detachment around the optic nerve, congestion of dilated blood vessels, tissue edema, and an increase in hypertrophic and degenerated astrocytes. Consistent with our results, a murine study showed that topical erlotinib adversely affected corneal epithelial histopathology [61]. Optic nerve histology in the TPP group was comparable to that of the healthy control group. Congestion was infrequently noted in the connective tissue blood vessels adjacent to the optic nerve. Cinici et al. showed that TPP significantly mitigated hyperglycemia-induced retinopathy [62]. Another study indicated that histopathological findings in retinal tissues resulting from drug-induced ocular toxicity in vivo were similar to those in normal tissues following TPP administration [63]. Cinici et al. noted the prophylactic advantage of TPP in vascular retinopathy, which may occur in individuals without diabetes who consume carbohydrate-rich diets [64]. Cicek et al.’s study on ocular ischemia demonstrated that TPP mitigated oxidative ocular damage, and our findings align with the existing literature [65].

## 5. Conclusions

Biochemical and histological analyses demonstrated that erlotinib caused oxidative and inflammatory damage in the rat optic nerve, which was greatly mitigated by TPP. These findings support TPP as a candidate neuroprotective adjunct against EGFR-inhibitor-related optic nerve injury. Additional preclinical research—establishing dose–response relationships and exposure levels while integrating functional assessments—alongside meticulously structured clinical trials is essential to ascertain translational significance.

### Limitations

Several limitations should be acknowledged. First, the single-dose erlotinib protocol restricted dose–response analysis; studies including numerous doses are necessary to more accurately define erlotinib toxicity and the protective window of TPP. Second, the lack of positive-control comparators (agents with established antioxidant/anti-inflammatory properties) constrains interpretability. Third, we measured pro-inflammatory cytokines but excluded anti-inflammatory mediators; including anti-inflammatory cytokines (e.g., Transforming growth factor-Beta [TGF-β], interleukin-10 [IL-10]) would elucidate the etiology of erlotinib-induced optic nerve injury. Fourth, direct quantification of oxidative DNA and protein injury was not performed; integrating markers such as 8-hydroxy-2′-deoxyguanosine [8-OHdG] and aconitase activity in future studies would offer a more mechanistic understanding of oxidative damage pathways. Fifth, a more comprehensive histopathological characterization of the optic nerve, including advanced staining and immunohistochemical methods, was also not undertaken in the present study. Such analyses would provide a more detailed assessment of demyelination, axonal degeneration, and cellular inflammatory responses, thereby allowing a clearer correlation between structural alterations and biochemical findings. Finally, functional assessments of visual performance (e.g., optokinetic tracking [OKT] and flash visual evoked potentials [VEPs]) were not feasible due to current infrastructural limitations; incorporating such techniques would strengthen the translational value of the findings.

## Figures and Tables

**Table 1:** Comparative evaluation of erlotinib and thiamine pyrophosphate regarding their impact on oxidant, antioxidant, and pro-inflammatory responses in the optic nerve of rats.

	Posthoc Test *p*-Values					
Group Comparisons	MDA	tGSH	SOD	CAT	IL-1β	TNF-α
HG vs. TPPG	0.847	0.933	0.824	0.367	0.806	0.764
HG vs. ERTG	<0.001	<0.001	<0.001	<0.001	<0.001	<0.001
HG vs. ERTPG	0.607	0.834	0.415	0.579	0.743	0.117
TPPG vs. ERTG	<0.001	<0.001	<0.001	<0.001	<0.001	<0.001
TPPG vs. ERTPG	0.201	0.494	0.102	0.036	0.254	0.016
ERTG vs. ERTPG	<0.001	<0.001	<0.001	<0.001	<0.001	0.002
F value	63.164	86.605	114.771	237.632	67.221	23.319
df (df1/df2)	3/20	3/20	3/20	3/20	3/20	3/20
*p*	<0.001	<0.001	<0.001	<0.001	<0.001	<0.001

**Footnotes:** Intergroup differences were first assessed using one-way ANOVA, and post-hoc pairwise comparisons were subsequently performed with Tukey’s Honestly Significant Difference (HSD) test. **Abbreviations**: HG: healthy group; TPPG: TPP alone group; TPP: thiamine pyrophosphate; ERTG: erlotinib alone group; ERTPG: erlotinib + TPP group; MDA: malondialdehyde; tGSH: total glutathione; SOD: superoxide dismutase; CAT: catalase; IL-1β: interleukin-1 beta; TNF-α: tumor necrosis factor-alpha; df: degree of freedom.

**Table 2:** Semi-quantitative evaluation of histopathological alterations in rat optic nerve tissue.

	Histopathological Grading Data		
Groups	Increase in Astrocyte Cell Population	Edema	Vascular Dilatation/Congestion
HG	0 (0–0)	0 (0–0)	0 (0–0)
TPPG	0 (0–0)	0 (0–0)	0 (0–0)
ERTG	2 (1–3)	2 (1–3)	2 (1–3)
ERTPG	0 (0–2)	0 (0–1)	0 (0–2)
Group comparisons	*p*-values		
HG vs. TPPG	1.000	1.000	1.000
HG vs. ERTG	<0.001	<0.001	<0.001
HG vs. ERTPG	0.150	0.085	0.005
TPPG vs. ERTG	<0.001	<0.001	<0.001
TPPG vs. ERTPG	0.150	0.085	0.005
ERTG vs. ERTPG	<0.001	<0.001	<0.001
KW	115.923	119.259	113.115
df	3	3	3
Total *n*	144	144	144
*p*-value	<0.001	<0.001	<0.001

**Footnotes:** Data are presented as median (minimum–maximum). Statistical analysis for inter-group comparisons was performed using the Kruskal–Wallis test. For post hoc multiple comparisons, Dunn’s test with Bonferroni correction was applied, and adjusted *p*-values are reported. Sample size for all groups: *n* = 6. **Abbreviations:** HG: healthy group; TPPG: TPP alone group; TPP: thiamine pyrophosphate; ERTG: erlotinib alone group; ERTPG: erlotinib + TPP group; KW: Kruskal–Wallis test; df: degree of freedom.

## Data Availability

The original contributions presented in this study are included in the article/Supplementary Materials. Further inquiries can be directed to the corresponding author.

## Supplementary Materials

The following supporting information can be downloaded at: https://www.mdpi.com/article/10.3390/biomedicines13112614/s1, Table S1. The assumption of normality for biochemical outcomes in rat optic nerve tissue was verified using the Shapiro–Wilk test. Table S2. The assumption regarding homogeneity of variances was tested in the datasets of MDA, tGSH, SOD, CAT, IL-1β, and TNF-α.
