# Supplementary material for: Protective Role of Thiamine Pyrophosphate Against Erlotinib-Induced Oxidative and Inflammatory Damage in Rat Optic Nerve
Source: Biomedicines. 2025 Oct 25;13(11):2614. doi: 10.3390/biomedicines13112614 (PMC12650006; doi:10.3390/biomedicines13112614)
Supplement: Supplementary file 1 [file biomedicines-13-02614-s001.zip › Supplemantary Table S1.pdf]

Supplementary Table S1. The assumption of normality for biochemical outcomes in rat optic nerve tissue was verified using the Shapiro–Wilk test

			Biochemical Variables					
		**Shapiro-Wilk**	**MDA**	**tGSH**	**SOD**	**CAT**	**IL-1β**	**TNF-α**
**Groups**	**HG**	**statistic**	0.893	0.971	0.973	0.908	0.948	0.975
		**df**	6	6	6	6	6	6
		**sig.**	0.334	0.900	0.912	0.424	0.728	0.924
	**TPPG**	**statistic**	0.977	0.952	0.928	0.906	0.889	0.919
		**df**	6	6	6	6	6	6
		**sig.**	0.933	0.755	0.564	0.414	0.312	0.495
	**ERTG**	**statistic**	0.955	0.965	0.933	0.957	0.974	0.926
		**df**	6	6	6	6	6	6
		**sig.**	0.779	0.858	0.605	0.799	0.918	0.549
	**ERTPG**	**statistic**	0.954	0.945	0.959	0.945	0.930	0.975
		**df**	6	6	6	6	6	6
		**sig.**	0.776	0.700	0.814	0.697	0.581	0.922

Footnotes: Since the levels of MDA, tGSH, SOD, CAT, IL-1β, and TNF-α met the assumptions of normal distribution, group comparisons were carried out using ANOVA.
Abbreviations: TPP: thiamine pyrophosphate; HG: healthy group; TPPG: TPP alone group; ERTG: erlotinib alone group; ERTPG: erlotinib + TPP group; MDA: malondialdehyde; tGSH: total glutathione; SOD: superoxide dismutase; CAT: catalase; IL-1β: interleukin-1 beta; TNF-α: tumor necrosis factor-alpha; df: degrees of freedom; sig: significance.
