# Supplementary material for: Protective Role of Thiamine Pyrophosphate Against Erlotinib-Induced Oxidative and Inflammatory Damage in Rat Optic Nerve
Source: Biomedicines. 2025 Oct 25;13(11):2614. doi: 10.3390/biomedicines13112614 (PMC12650006; doi:10.3390/biomedicines13112614)
Supplement: Supplementary file 1 [file biomedicines-13-02614-s001.zip › Supplemantary Table S2.pdf]

Supplementary Table S2. The assumption regarding homogeneity of variances was tested in the datasets of MDA, tGSH, SOD, CAT, IL-1β, and TNF-α

	**Biochemical Variables**					
	**MDA**	**tGSH**	**SOD**	**CAT**	**IL-1β**	**TNF-α**
**Levene's statistic**	0.288	0.100	0.827	0.858	0.953	2.256
**df1**	3	3	3	3	3	3
**df2**	20	20	20	20	20	20
**sig.**	0.834	0.959	0.495	0.479	0.434	0.113

Footnotes: Since the homogeneity of variances assumption was satisfied, post-hoc comparisons of MDA, tGSH, SOD, CAT, IL-1β, and TNF-α were performed using Tukey's honestly significant difference (HSD) test.

Abbreviations: MDA: malondialdehyde; tGSH: total glutathione; SOD: superoxide dismutase; CAT: catalase; IL-1β: interleukin-1 beta; TNF-α: tumor necrosis factor-alpha; df: degrees of freedom; sig: significance.
